# Who is in crisis?

**DOI:** 10.1093/bjs/znaf134

**Published:** 2025-07-15

**Authors:** Dion G Morton, Abdul Ghaffar

**Affiliations:** Department of Allied Health, University of Birmingham, England; Department of Allied Health, University of Birmingham, England; Community Health Science, The Aga Khan University, Karachi, Pakistan

The WHO has come under huge financial pressure following substantial and sudden budget cuts by the United States^1^, which have been compounded by some other countries following suit. These cuts were put in place without warning. They have put aid programmes across the world in jeopardy, and patients and populations are already suffering. These constraints have also adversely affected the development of surgical care across low- and middle-income countries (LMICs). One real fear is that with contraction of Overseas Development Assistance (ODA), development of global surgery will fall even further off the radar, being seen as inessential for the development of public healthcare systems. This year has already seen the progress of a key World Health Assembly resolution 76.2^2^ (Emergency, Critical and Operative care) stall, as the WHO undergoes dramatic reorganization.

The future for the development of surgical care was already challenging, but some green shoots are appearing in the surgical arena, often driven by surgical teams from across the world. The Health Policy for Surgery 2025–35 meeting was held in Geneva in May, hosted jointly by the *BJS* and the *Lancet*. Here a plan for the next 10 years was laid out. It reported on data collected by the global network of surgical teams, coordinated through the NIHR Global Surgery Unit^3^. Nurses, anaesthetists, allied health professionals, public health leaders, and surgeons have come together to form a community of over 40 000 clinical staff in 2000 hospitals and 120 countries to improve the care of patients across the world. This is now providing a robust and agile platform for the collection of accurate, real-time data on surgical activity and outcomes. Without this information, governments and development agencies will not invest in surgical services.

The health policy plan for surgery^4^, using patient-level data, highlighted and explained the progress made in the 10 years since the Lancet Commission on Surgery^5^. The report identified challenges—not least that the unmet need for surgery has actually grown in that time. This is not only because of failing services, but also due to the increasing needs of an ageing global population, in addition to the demands created by global crises upon already constrained services. Of course, the greatest need and largest gap is seen in the world’s poorest countries.

It is increasingly recognized that the surgical service is essential for an effective and resilient health system and the policy paper describes how surgical services can strengthen the systems across the hospital as well as pathways of care from the community to the healthcare facilities. Moreover, a new modelling exercise using the clinical network data has estimated the financial loss from lack of surgery for adult cancers to be in excess of $80 billion every year. Such data are certainly key for governments to prioritize and allocate funding.

The plan for the next 10 years will utilize the global surgical network to provide patient-level data through snapshot audits such as AlliGatOr^6^, which is currently being completed. This is being combined with surveys of hospital and primary care systems to identify the current patient need and for the clinical network to identify solutions. Interventional trials such as the task shifting trial, TIGER^7^, can then test the solutions on the same global platform. Surgical teams have an essential role in this process. This work is empowered by high- and low-income country teams working together. High-income country teams can provide contemporaneous patient data and much-needed research experience. Contemporaneous data can highlight what is currently achievable. Research experience ensures the delivery of high-quality evidence. Prioritization of health spending is a worldwide problem affecting all countries and governments. Identifying cost-effective treatments, learning from each other, and not repeating the mistakes of established surgical services are some of the lessons that can be learnt from these global initiatives. Findings from these studies benefit all patients.

Development of surgical services is essential for strengthening national and subnational health systems. Development of these services will increase access to the needy, reduce the unmet need, save lives, improve quality of life, and save billions of dollars lost in productivity. Investing in strengthening surgical services is time critical and needs the support of clinical as well as health systems leadership.
